# Thinking in opposites improves hypothesis testing performance in Wason’s rule-discovery task

**DOI:** 10.3758/s13421-025-01691-3

**Published:** 2025-02-26

**Authors:** Erika Branchini, Ivana Bianchi, Roberto Burro

**Affiliations:** 1 Department of Human Sciences, University of Verona, Lungadige Porta Vittoria 27, 37129 Verona, Italy; 2 Department of Humanities (Section Philosophy and Human Sciences), University of Macerata, Corso Garibaldi 20, 62100 Macerata, Italy

**Keywords:** Thinking in opposites, Inductive task, Hypothesis testing, Wason’s rule-discovery task

## Abstract

We investigate whether hypothesis testing can be improved by a simple prompt to “think in opposites,” a strategy suggested by a growing body of literature as being beneficial in various reasoning and problem-solving contexts. We employed Wason’s rule-discovery task and designed three experimental conditions: training that prompted an analysis of the properties characterizing the initial seed triple, training that prompted the same analysis but subsequently required the identification of the opposites of each property for use in the testing phase, and a no-prompt condition. Thinking in opposites nearly doubled the success rate and led to a more frequent discovery of the rule on the first attempt. This improved efficacy was due not to the testing of more triples but to less reiteration of the same hypothesis and a greater awareness of the ascending-descending critical dimension. We discuss how thinking in opposites appears to stimulate counterfactual thinking, with respect to previous literature.

## Introduction

Since Popper (1962), falsification in hypothesis testing in science has acquired importance. Popper emphasized that scientists should strive to disconfirm their theories rather than merely confirm them, as accumulating facts that align with the theory predictions does not counter a single case that contradicts those predictions. Using Popper’s classic example, to prove that “all swans are white,” one should seek a black swan, rather than collect white swans. As several studies in the psychology of reasoning have shown, testing the theory that all swans are white by looking for white swans is easier and somehow more intuitive for humans than looking for black swans. The bias of seeking cases that confirm a hypothesis rather than disconfirm it has been termed “confirmation bias” (McKenzie, 2004; Nickerson, 1998). Confirmation bias is a general phenomenon that has emerged in hypothesis testing across various contexts. For instance, it has been found that, after having taken a decision, individuals exhibit reduced sensitivity to subsequent contrasting information (Bronfman et al., 2015), tend to seek choice-consistent evidence (e.g., Bressler et al., 2018; Snyder & Campbell, 1980; Talluri et al., 2018), and discredit information that contradicts their held hypotheses (e.g., Kappes et al., 2020; Ross & Lepper, 1980). In the following sections, we discuss this bias specifically in relation to Wason’s rule-discovery task, as our study tested the effect of a prompt to think in opposites on improving hypothesis testing performance using this specific task.

Before detailing Wason’s task and the hypotheses underlying the study, we would like to highlight that hypothesis testing is ubiquitous in human thinking. Not only do scientists form hypotheses about specific phenomena and strive to determine their validity while revising them in light of inconsistent data discovered, but doctors also generate hypotheses based on reported symptoms and diagnostic evidence to suggest treatments, adjusting both their diagnostic hypotheses and treatments based on follow-up evidence. Similarly, car mechanics diagnose likely damage based on initial information (reported or directly observed) and adjust their hypotheses based on feedback from subsequent tests. Further, individuals use evidence gathered from everyday experiences to generate hypotheses about social relationships (e.g., “when I express a contrasting opinion, even kindly, they become upset”; “they are very generous people”), and then test and confirm or revise these hypotheses based on further experiences. Thus, we are not dealing with an uncommon or technical thinking process, but rather with a widespread type of reasoning common in our daily lives.

Among the various tasks used to study hypothesis testing processes, Wason’s (1960) 2-4-6 rule-discovery task is a classic. In this task, participants are asked to discover a rule (known only to the experimenter) that generates sequences of three numbers (triples). A seed triple (2-4-6) is provided as an example of the rule. Participants are instructed to generate as many test triples as necessary to discover the rule. For each submitted test triple, they receive feedback from the experimenter, who confirms or denies the triple's conformity to the rule in mind. When participants believe they have identified the rule, they announce it. The rule to be discovered is “three ascending numbers.”

While research on this task has been out of fashion for over a decade, this paradigm has not been exhausted in terms of what it can reveal about the nature of hypothesis testing and how it might be facilitated (as we will see in the next section, the task typically has a very low solution rate). In the present paper, we revive the use of this task to test a novel hypothesis – that stimulating reasoners to “think in opposites” might improve the solution process. The rationale for exploring whether this prompt might be beneficial is the growing empirical evidence suggesting that opposites are central to imagining alternatives in various reasoning processes (Bianchi & Branchini, 2023; Branchini et al., 2021; Byrne, 2018; Fitzgibbon et al., 2019), including problem-solving (Bianchi et al., 2020; Branchini et al., 2023, 2015, 2016). We wondered whether the same suggestion might also be beneficial in hypothesis testing, and used Wason’s task to test it.

Some initial positive indications of this prompt in Wason’s task emerged in a previous study (Branchini et al., 2023), but its experimental design did not allow the authors to determine whether the effectiveness of the prompt depended specifically on the use of opposites or whether it was induced by the request to focus analytically on the properties of the initial seed triple, which constituted part of the instructions provided to participants. The study design presented in this paper allows us to clarify this point. We expected that the suggestion to test triples with opposite properties, compared to the seed triple, might ultimately stimulate participants to adopt a disconfirmatory strategy, rather than a confirmatory one. The rationale for expecting this benefit is that several studies have indicated that when asked to identify the opposite of a stimulus – a figure (Bianchi & Burro, 2023; Bianchi & Savardi, 2008), a gesture (Bianchi et al., 2014), or a simple transformation process (Bianchi et al., 2022; Capitani et al., 2020) – people tend to select a figure, gesture, or process that is opposite to only *one* aspect. In Oaksford and Chater’s (1994a) iterative counterfactual model described below, alternative counterfactual hypotheses are generated by varying one aspect at a time (Tschirgi, 1980; see also Gale & Ball, 2012). Thinking in opposites thus appears potentially fruitful, as it aligns well with the idea of finding an evident counterfactual. Notably, in Popper's example, a *black* swan is chosen as counterfactual evidence that not all swans are *white* (while any non-white swan would logically fit the requirement).

Before presenting our study, we briefly outline the hypothesis generation and testing processes typically used to solve Wason's rule-discovery task. We then present existing studies that suggest the effectiveness of prompts based on contrast/opposition in reasoning tasks, particularly in facilitating Wason’s task, which forms the background of our research.

### The hypothesis generation and testing processes in Wason’s rule-discovery task (the 2-4-6 task)

Wason’s 2-4-6 task is a classic laboratory task used to study hypothesis testing. Wason intended it to simulate a miniature scientific problem (Wason, 1960, p. 139). The universe, in this case, consists of all possible sets of triples, divided into a set that fits the rule (like the white swans in Popper’s example) and another set that does not (corresponding to the non-white swans). Participants begin with an initial guess, suggested by the seed triple 2-4-6, transform it into a hypothesis, and then submit triples they expect to either conform to the subset of events that fit the rule (a confirmatory process or positive hypothesis test) or to the complementary set of events that do not fit it (a disconfirmatory process or negative hypothesis test).

This task allows researchers to investigate not only how reasoners build their generalizations from the available data (i.e., the initial triple and the feedback received in response to the test triples submitted) but also how they generate tests of their own hypotheses (Klahr & Dunbar, 1988). Although the task may appear simple, only about 20% of participants solve it correctly on their first attempt (Cherubini et al., 2005; Sperber et al., 1995; Tukey, 1986; Van der Henst et al., 2002; Wason, 1960; Wharton et al., 1993).

The low success rate in Wason’s task is due to participants’ tendency to adopt a positive test strategy (Cherubini et al., 2005; Evans, 2016; Hegarty, 2017; Kareev et al., 1993; Klayman & Ha, 1987, 1989; Rossi et al., 2001; Spellman et al., 1999; Vallée-Tourangeau et al., 1995). Klayman and Ha (1989) describe the positive test strategy as a general default heuristic that, while serving the basic goal of determining whether or not a hypothesis is correct in many situations, can lead to errors in particular circumstances, as in the case of any all-purpose heuristic. “According to this strategy, you test a hypothesis by examining instances in which the property or event is expected to occur (to see if it does occur), or by examining instances in which it is known to have occurred (to see if the hypothesized conditions prevail)” (Klayman & Ha, 1989, p. 212). Adopting a positive hypothesis test strategy in Wason’s task means submitting triples expected to be in the target set that verifies the rule (i.e., a white swan), rather than in the non-target set (i.e., non-white swans). Therefore, if, for example, the seed triple 2-4-6 leads participants to guess that the rule is “three ascending even numbers,” they might test whether 4-6-8 or 12-14-16 (which they expect to fit the rule) receive positive feedback from the experimenter.

The problem in Wason’s task is that the seed triple 2-4-6 orients the majority of participants towards rules that are more specific than the rule to be discovered, which is simply “ascending numbers.” Various experimental studies have demonstrated that while participants notice many properties common to the original and generated triples (such as “being in ascending order by intervals of two”), they rarely tend to note the characteristic of the triples “being arranged in ascending order” (Cherubini et al., 2005; Gorman, 1992; Gorman et al., 1984, 1987; Mynatt et al., 1977, 1978; Shafir, 1993; Tweney et al., 1980; Vallée-Tourangeau et al., 2005; Van der Henst et al., 2002; Wason, 1960; Wetherick, 1962). According to Cherubini et al. (2005), this blindness stems from participants perceiving the co-occurrences of properties (e.g. “being arranged in ascending order” and “being arranged in intervals of two”) as constituting a salient structure. Reasoners intuitively believe that this structure (of co-occurrence) is more informative than one involving the presence of simply one property; this explains their tendency to neglect the rule of “being arranged in ascending order,” instead of considering it in combination with another property.

If that is the premise (and, for example, people think of “even ascending numbers” or “numbers increasing by two” as an initial hypothesis), using a positive hypothesis test strategy means they will never discover that the rule is incorrect because all the examples they submit of triples receive positive feedback, which reinforces their hypothesis (Cherubini et al., 2005; Cooper et al., 2022; Dasgupta et al., 2017; Gale & Ball, 2012; Hayes & Heit, 2018; Hayes et al., 2019, 2023; Hegarty, 2017; Klayman & Ha, 1987; Sauerland et al., 2020). In other words, when the hypothesis formulated by reasoners is narrower than the correct one, using a positive testing strategy makes it impossible to encounter negative feedback. In this case, it would be crucial to attempt a negative hypothesis test (i.e., submit a triple formed by “odd ascending numbers,” e.g., 1-3-5, if they hypothesize that the rule is “even ascending numbers,” or submit a triple that “does not increase by 2,”” e.g., 2-4-7, in the case they hypothesize that “numbers increasing by two” is the rule).

Wason deliberately designed his task so that the hypotheses that strike reasoners first are narrower than the correct rule. However, not all situations involving hypothesis testing are like this. In other situations, the set of instances that fit the rule to be found may be as broad as the set of instances that fit the hypothesis the reasoner has in mind, or may be narrower compared to it, or disjointed from it. In any of these three circumstances, using a positive test strategy can lead to the correct identification of the rule, as the strategy can produce conclusive falsification (see Klayman & Ha, 1987, pp. 213–215, for a detailed analysis). Because in natural situations one can find oneself in any of the four conditions presented (and in three of them, the discovery of the non-white swan is possible), the use of a positive test strategy cannot be condemned per se, as it is functional and successful in many cases. However, in Wason’s task, it is not, which is why the task has been extensively used to investigate hypothesis testing and why we employed it in our study.

Oaksford and Chater’s iterative counterfactual model (1994a, b) – a refinement of Farris and Revlin’s model (1989a, 1989b) – provides a different perspective on the hypothesis generation and verification process, which is also relevant to our study. According to this model, hypothesis generation and verification occur through an iterative process of producing working and counterfactual hypotheses to explore the boundaries of a rule. This means that participants engaged in Wason’s task while searching for features common to the seed triple and the test triples they must submit, generate an initial working hypothesis (H), for example, “even increasing numbers by intervals of two.” Based on this first hypothesis, they produce other triples to be tested in a confirmatory manner, which can lead to positive feedback. Subsequently, participants generate an alternative or counterfactual hypothesis (H1) by varying one aspect at a time (Tschirgi, 1980). H1 differs from the first hypothesis by only one dimension, for example, in terms of “*odd* increasing numbers by intervals of two.” The triple generated by the counterfactual hypothesis can also receive positive feedback (as would indeed happen in Wason's task, since both even and odd ascending numbers fit the rule). However, it can also receive negative feedback if, for instance, the alternative counterfactual hypothesis (H1) “even *decreasing* numbers by intervals of two” is proposed. According to the iterative counterfactual model, the continuous process of generating and testing both working and counterfactual hypotheses leads to the discovery of the correct rule. Interestingly, in this model, the application of a positive test strategy is not an obstacle. Indeed, once reasoners have identified the alternative hypothesis they wish to test (e.g., “*odd* increasing numbers by intervals of two” or “even *decreasing* numbers by intervals of two”), they are expected to submit triples that conform to it (e.g., 1-3-5 or 5-7-9 to test the first H1, or 6-4-2 or 10-8-6 to test the second H1). Submitting triples that conform to the hypothesis they have in mind is straightforward, as demonstrated by the “positive test bias.” What seems critical, therefore, is stimulating participants to generate alternative counterfactual hypotheses in their minds. This study, along with previous ones reviewed in the next section, aimed to achieve this.

### The facilitating factors of contrast/opposition in Wason’s rule-discovery task

Several studies have investigated the mechanisms or factors that might facilitate the discovery of the correct rule in Wason’s rule-discovery task (Branchini et al., 2023; Gale & Ball, 2006, 2012; Gorman & Gorman, 1984; Gorman et al., 1984, 1987; Rossi et al., 2001; Tukey, 1986; Tweney et al., 1980; Vallée-Tourangeau et al., 1995; Wharton et al., 1993). Some of the facilitatory strategies tested in these studies emphasize the importance of contrast or opposition through instructions, cues, or training programs (Branchini et al., 2023; Gale & Ball, 2012; Gorman & Gorman, 1984; Gorman et al., 1984, 1987; Rossi et al., 2001). This literature is particularly relevant to our paper.

For example, Rossi et al. (2001) found a beneficial effect of presenting the initial triple 2-4-6 as a *counter-example* of the rule the experimenter had in mind (i.e. “decreasing numbers’) rather than as an *example* of the rule (i.e. “increasing numbers’) – this last condition was used as a control condition. In the counter-example condition, participants formed their hypotheses based on the salient features of the initial triple, and frequently hypothesized that the rule to be discovered was “ascending *odd* numbers” (which is also the most common initial rule they announced). Accordingly, they tested triples such as 3-5-7, which received negative feedback, since the experimenter’s rule was “decreasing numbers.” However, in the counter-example condition, participants produced, on average, almost twice as many test triples before announcing the first rule compared to the example condition, and the number of negative feedback instances they collected was more than four times higher than in the example condition. Due to the negative evidence produced, participants progressively eliminated the “increasing triples” hypotheses, enhancing their probability of discovering the experimenter’s rule, which was indeed found in more than 50% of cases on the first attempt.

Gorman and colleagues (1984, 1987) encouraged participants to use a disconfirmatory strategy (and compared this performance with that found when a confirmatory strategy or no strategy at all was employed) by inviting them to test not cases they expected would confirm the hypothesis (positive feedback) but those they anticipated would receive negative feedback. For instance, in a game task where participants were asked to find a rule underlying the classification of some cards as correct instances of a rule, participants in the disconfirmatory condition were invited to test their guesses “by deliberately playing cards that you think will be wrong” and to “look at previous wrong cards and see if they follow a pattern.” Conversely, in the confirmatory conditions, they were asked to concentrate on playing cards “that you think will be correct” (Gorman et al., 1984, p. 3). The results are controversial, as the strategy proved beneficial in the card condition (Gorman et al., 1984), while in the original Wason’s 2-4-6 task, positive effects were found not in the original version of the task, but in its DAX-MED version (Gorman et al., 1987).

The DAX-MED dual-task (Tweney et al., 1980) is the most successful manipulation of Wason’s classic task reported in the literature, effectively stimulating the use of a counter-example procedure. In this task, participants must discover two complementary rules: one (named DAX) is Wason’s original “increasing number” rule; the alternative rule (named MED) is “all other number triples.” Gale and Ball (2009, 2012) demonstrated that the usefulness of a presented MED exemplar depended on whether it helped reasoners establish a helpful “contrast class” in opposition to the 2-4-6 DAX exemplar. They found significant improvements in participants’ performance when the seed triple 2-4-6 was offered as an example of DAX and the seed triple 6-4-2 was provided as an example of MED (with a solution rate of around 75%). Participants performed better with this condition than in conditions where the triples 4-4-4 (around 20% success) or 9-8-1 (around 34% success) were used as examples of MED. These results are presented by the authors as evidence of the role that contrast classes play in generating useful alternative hypotheses, and are discussed particularly concerning Oaksford and Chater's (1994a, b) iterative counterfactual model. Indeed, the seed triples 2-4-6 and 6-4-2 contrast along the “increasing-decreasing” dimension, which is relevant to the solution. The other pairs contrast along either a non-useful contrast class (ascending numbers vs. equal numbers for the pair 2-4-6 and 4-4-4) or along multiple dimensions (the pair 2-4-6 and 9-8-1).

In a different framework, but based on somewhat similar premises, Branchini et al. (2023) tested whether training participants to list all the properties of the initial seed triple, identify their respective opposites, and finally use these opposites in the hypothesis testing phase might improve solution rates. The training was derived from a similar process that had been successfully applied to solve visual-spatial insight problems (Bianchi et al., 2020; Branchini et al., 2016). The positive results found are discussed by the authors as further evidence that reasoning in terms of contrast might positively support the generation of alternative hypotheses – in line with Gale and Ball (2012) and somehow in continuity with what was indirectly suggested by Oaksford and Chater (1994a, b). However, since the study had some limitations, no solid conclusions could be drawn from the existing data.

### The study

Our study aimed to better understand the efficacy of training that encourages thinking in opposites while solving Wason’s rule-discovery task. In the work of Branchini et al. (2023), the training condition was matched only with a control condition without training. The positive results of their experiment may have depended simply on the prompt to focus on different properties of the seed triple, one at a time, which was kept implicit in the training process; delving into this aspect (and not the use of opposites per se) might have improved the success rate.

The current study was designed to clarify this aspect and to better understand the specific impact (in terms of process, not only of success rate) of using opposites. We designed three conditions: training based on opposites (TO), with three phases (1. Identification of the properties of the original triple, 2. Identification of the opposite of each property, and 3. Production of a triple based on the identified opposite); training based on listing the triple properties (TP), consisting of two phases (1. Identification of the properties of the original triple and 2. Production of a triple based on the identified property); and a control condition (C), where no training was offered. We compared our participants’ performance across the three conditions with respect to the following indices: success rates, effectiveness of attempts in terms of both solution times and finding the solution on the first attempt, number of test triples submitted, identification of the critical dimension (ascending-descending), identification of two other relevant dimensions (regular-variable intervals between numbers and odd-even numbers), and flexibility of the hypotheses tested. As thinking in opposites was the focus of our experiment, we expected the following predictions to hold (or at least some of them):

- *Success rates*: More correct solutions in the TO condition compared to the other two conditions would indicate the training efficacy.
- *Effectiveness of attempts*: We expected more correct solutions being submitted on the first attempt in the TO condition (participants had three attempts to announce the rule), even if they did not necessarily require a shorter response time since identifying opposites is time-consuming (at least as time-consuming, but possibly more so, than overtly listing the properties of the seed triple in the TP condition).
- *Number of triples submitted*: No specific directional prediction was made regarding this hypothesis, as the two training conditions might support a more extensive analytical exploration and testing of the seed triple properties; conversely, the reiteration of triples testing the same hypothesis might lead to an overall equal or more triples tested in the control condition. Instead of analyzing the total number of triples produced by each participant (which would have been influenced by the number of attempts made – if a participant discovered the rule on their first attempt, they would not submit further triples), we analyzed the average number of triples produced before each attempt. This indicates the average length of the series of test triples prior to attempting to guess the rule.
- *Flexibility* of participants’ hypothesis testing process: This aspect complements the previous index regarding the number of triples submitted and helps understand it. We expected reduced emphasis on testing the same hypothesis and reiterating similar testing triples in the TO and TP conditions compared to the C condition, due to the suggested procedure.
- *Type of triples submitted*: We expected the impact of the TO training to manifest particularly in the type of triples tested, rather than in their overall number. Specifically, we wondered whether the TO condition would support the identification of the critical dimension “ascending-descending” and the other two relevant dimensions (“constant-variable intervals” between numbers and “odd-even” numbers). If participants submitted at least one triple that contrasted the seed triple or one of the previously submitted test triples by using a descending rather than an ascending sequence (e.g., 6-4-2, 30-20-10), we considered this an indication that they explored the “ascending-descending” dimension. If a participant submitted at least one triple that varied the interval between numbers, we considered it as an indication that they explored the “constant-variable intervals” dimension (e.g., 2-4-10, 100-8-2). If they submitted at least one triple that contained odd numbers (e.g., 3-5-7, 5-9-21), we considered this as an indication that they were exploring the “odd-even” dimension. Of course, triples could contain a combination of these dimensions, as shown by some of the examples cited above in parentheses (e.g., 100-8-2, which varies both the intervals and the ascending-descending dimensions). We classified these cases accordingly, for all the dimensions involved (i.e., participant tested the “ascending-descending” dimension, participant tested the “constant-variable intervals” dimension).

## Method

### Participants

A total of 180 persons (88 males and 92 females; *M*_age_ = 30.11 years, *SD* = 13.57 years) participated in this study, recruited from various psychology classes. Those who volunteered to participate signed an informed consent form and received no credits for their participation. This study adhered to the ethical principles of the Declaration of Helsinki (World Medical Association, 2013) and was approved by the Ethics Committee of the University of Macerata (prot. n. 37413/2023).

### Procedure

Each participant was randomly assigned to one of the three conditions (i.e., 60 participants for each condition). The standard version of Wason’s rule-discovery task was used for all conditions. Participants were informed that they had to discover a rule (that the experimenter had in mind) governing the production of triples of numbers. They were told they would be given an initial triple as an example of the rule and that they should test as many triples as needed to discover the rule, receiving feedback (“yes, it conforms to the rule” or “no, it does not”) for each submitted test triple. When participants believed they had conducted enough tests and found the rule, they had to articulate it. If the rule was correct, the task ended; if it was not, they continued submitting test triples and had two more chances to discover the rule. Three attempts were given in total.

The experimenter read these instructions aloud, which were also printed on a paper given to participants, and asked whether the task was clear. If so, the seed triple 2-4-6 was provided. The solution time was calculated from the moment the seed triple was revealed until the end of the task (for success, or after the third unsuccessful attempt).

Participants assigned to the C condition were asked to solve Wason’s task without any suggestions regarding the strategy to be used. They had to write down the test triples as they submitted them, the feedback received from the experimenter, and the rule when they announced it.

Meanwhile, participants assigned to the other two training conditions (TO and TP) underwent a training session (following Branchini et al., 2023) before engaging with Wason’s task. A triple of bi-dimensional figures was used to exemplify the steps of the training, instead of a triple of numbers, to avoid mentioning properties that could subsequently be relevant to the solution of Wason’s task. Thereafter, these participants were invited to apply the procedure learned in the subsequent task, i.e., Wason’s 2-4-6 task. The complete texts of the TO and TP training – printed on two sheets of paper, given to the participants and read out aloud by the experimenter – are reported in Appendix 1. In the TP condition, participants were asked to identify all the properties of the original set of figures (or numbers) that they could notice, one at a time. After identifying a property, they had to write it down on the response sheet and use it to generate a test triple. In the TO condition, participants began with the same step as in TP; that is, they were asked to identify the properties of the seed triple one at a time, but in this case, they were instructed to write down not only the property but also its opposite. They were then invited to use the opposite to generate their test triples. In both the TO and TP conditions, participants were asked to write the test triple and the feedback received from the experimenter on the response sheet and to continue testing triples by applying the same procedure, until they felt they had discovered the rule.

No time limit was set for the task. On average, it took less than 15 min to complete it in all three conditions.

### Materials

Each participant was given a booklet consisting of two sheets, one with their personal information (age, gender, and informed consent), and the other that presented Wason’s task. The seed triple 2-4-6 was printed at the top of a third sheet (which was given by the experimenter to participants after reading the instructions and verifying that the task was clear) followed by three tables, one for each possible attempt. The tables comprised three columns and 15 rows. In the first column, participants reported the test triples in the order they submitted them; in the second column, they noted either the property on which they were focusing (in the TP condition) or the opposite pair of properties on which they were focusing (in the TO condition); in the third column, they reported the feedback received from the experimenter after submitting their test triple. A row was left blank at the end of each table for participants to note down the announced rule and the time of the announcement. For participants in the C condition, the central column was not included.

### Data analyses

The effects of the three conditions (TO, TP, and C) on the various dependent variables under study were analyzed using linear models (LMs), generalized linear models (GLMs; both LMs and GLMs were calculated using the stats R-package, version 4.4.1, R Core Team, 2024) and generalized linear mixed models (GLMMs, lme4 R-package, version 1.1–35.3; Bates et al., 2015). Thereafter, the analysis of deviance and Bonferroni post hoc tests were applied to the results of LMs, GLMs, and GLMMs. The power of each analysis was tested using the WebPower R-package (version 0.9.4; Zhang & Mai, 2018).

## Results

In the following sections, we describe the results related to each of the hypotheses tested in this study. While the calculated minimum effect size (expressed with the f2 index) was 0.129, considering a maximum of two predictors and referring to our sample, our a posteriori power analysis revealed a power of 0.993 (alpha 0.05). Meanwhile, our a priori power analysis indicated that a sample of 78 participants was required to achieve a power of 0.8 (alpha 0.05).

### a) Success rate in the three conditions

**Fig. 1:**
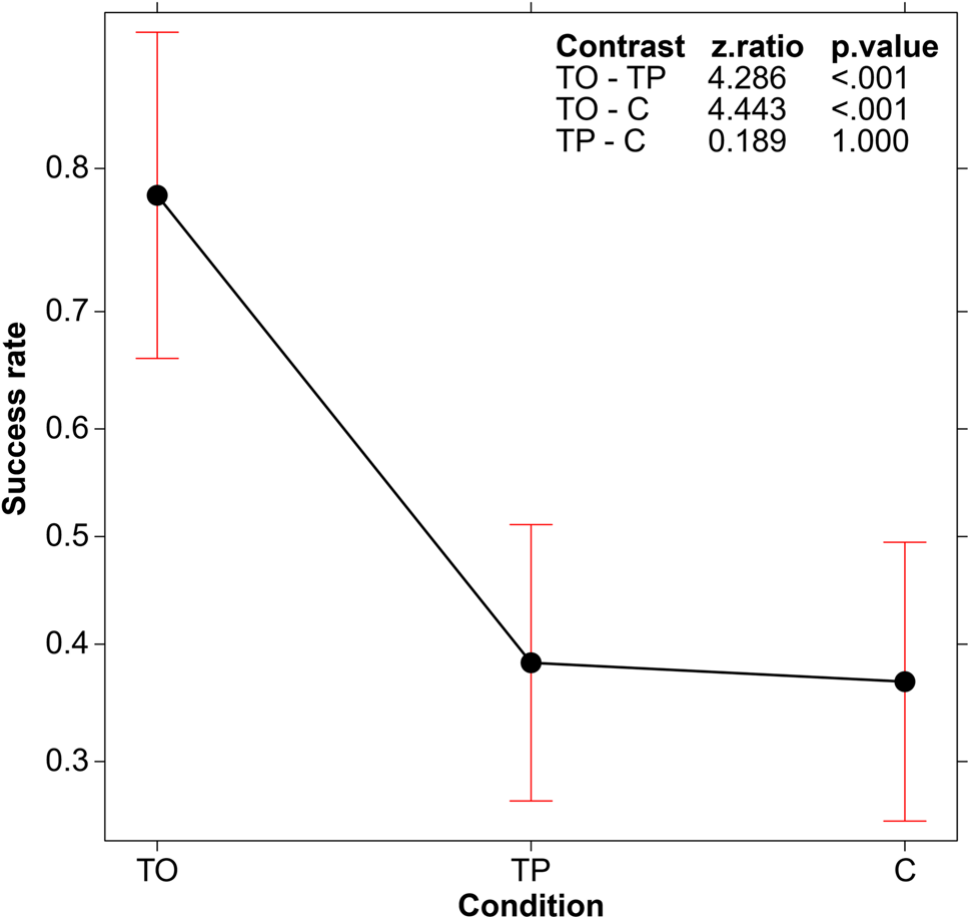
Main effect of correct solutions (proportion of participants who found the correct solution) in the three conditions – Training based on opposites (TO), Training based on listing the properties (TP), and Control (C) – and post hoc tests. Error bars represent the 95% confidence interval

A GLM (logit link function, binomial family) was used to study the proportion of participants who found the correct solution (to the total number of participants involved in the study in the TO, TP, and C conditions, respectively). The analysis revealed a significant difference in performance in the three conditions (*χ*^*2*^(2, *N* = 180) = 27.986, *p* < .001). As shown in Fig. 1, the post hoc tests confirmed that the proportion of correct solutions was significantly higher in the training condition based on opposites (TO) compared to the other two conditions (which did not differ from each other in terms of correct solutions). While 78.33% of participants found the correct solution in the TO condition, only 38.33% and 36.66% found it in the TP and C conditions, respectively.

### b) Discovery of the correct solution in terms of response times and the three possible attempts

**Fig. 2:**
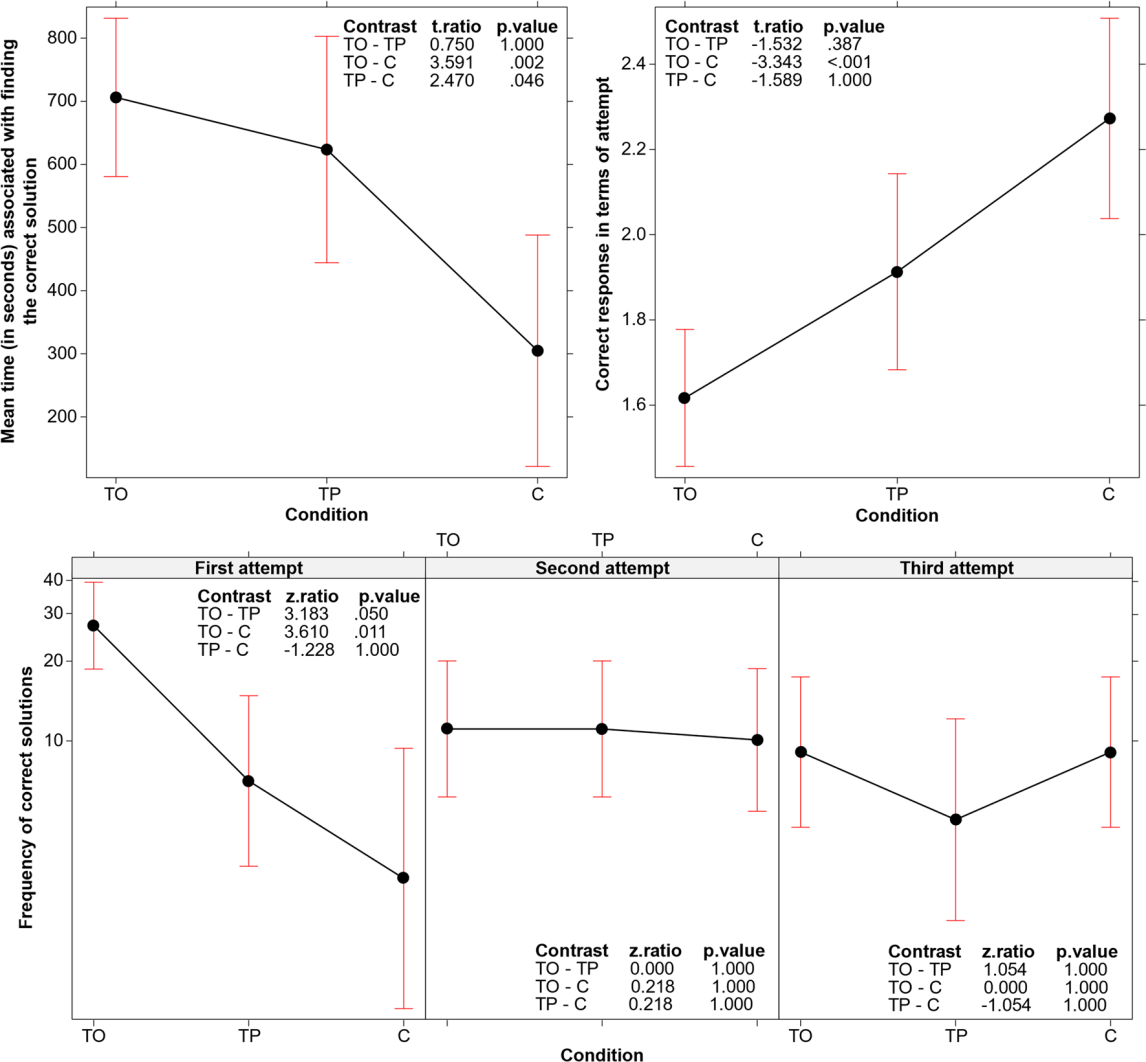
A **Top left panel:** Main effect of response time associated with the correct solution in the three conditions. **Top right panel:** Main effect of the attempt upon which the correct solution was found in the three conditions (average value; participants had a maximum of three attempts at their disposal). **Bottom panel:** Interaction between the attempt upon which the solution was found and condition. In all graphs, error bars represent the 95% confidence interval

To conduct the analyses reported in this section, we focused exclusively on the subset of participants who could find the correct solution. We wondered whether differences in the three conditions emerged concerning the time needed to find the correct solution (Fig. 2, top left panel) and in terms of the attempt upon which the solution was found, that is, did the participants succeed on the first, second, or third attempt (Fig. 2, top right panel)?

An LM on the response times associated with finding the correct solution in the three conditions proved significant (*F*(2, 89) = 6.541, *p* = .002). In both training conditions (TO and TP), on average, the correct solution took longer to arrive than in the control condition, indicating that the solution process took more time in the two training conditions (Fig. 2, top left panel). This could be attributed to participants needing time to identify and list the properties focused on and used for triple generation in TP, and the properties focused on and their opposites in TO. The identification of the properties (in TP) and of the properties and their opposites (in TO) was part of the procedure used to generate each triple and contributed to the total solution time recorded.

A second LM was conducted after recoding each correct response in terms of the attempt (i.e., first, second, or third) when it was articulated. This LM also proved significant (*F*(2, 89) = 5.702, *p* = .004). Particularly, the results (Fig. 2, top right panel) revealed that the solution, on average, was found earlier in TO than in C. TP was found to be in an intermediate position regarding C and TO (with no significant difference found between TP and C, or between TP and TO).

A GLM (log link function, Poisson family) was conducted to study the frequency of correct responses given on the first, second, and third attempts across all three conditions. The main effect of condition was significant (*χ*^*2*^(2, *N* = 180) = 12.288, *p* = .002), confirming what was shown in Fig. 1, although in this case with frequencies of responses rather than proportions of correct responses (i.e., participants found more correct solutions in TO than in the other two conditions). The more interesting result of this GLM is that the interaction between condition and attempts was also significant (*χ*^*2*^(2, *N* = 180) = 15.172, *p* = .004). As shown in the bottom panel of Fig. 2, it was at the first attempt that the advantage of TO became noticeable compared to C and TP. We found no differences among the three conditions when focusing on the second and third attempts.

### c) Number of triples submitted

**Fig. 3:**
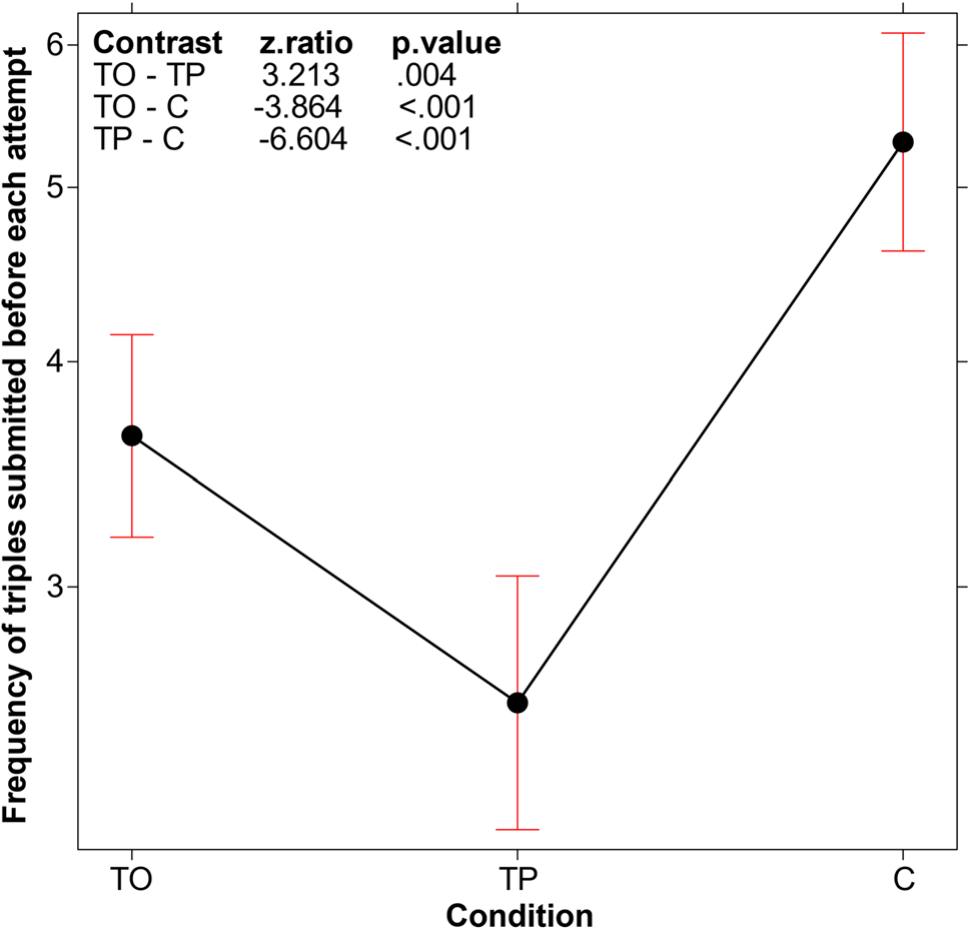
Main effect of the number of triples (average number of triples submitted before each attempt to announce the rule). Error bars represent the 95% confidence interval

A GLMM (log link function, Poisson family with condition as fixed effects and participants as random effect) was used to analyze the average number of triples submitted by participants prior to the rule announcement. This analysis aimed to assess the average number of triples submitted independently of the attempt number. The main effect of condition emerged (*χ*^*2*^(2, *N* = 180) = 44.325, *p* < .001), with the average number of triples being significantly higher in the C condition than in the TO and TP conditions (Fig. 3).

### d) Flexibility

We explored how flexible (or conversely, fixated on the same properties) the participants were in choosing the triples to be tested before each attempt. The triples were classified by two independent raters. Inter-rater agreement, assessed using Cohen's kappa coefficient, was good at 0.87. Disagreements were resolved through discussion between the two raters.

**Fig. 4:**
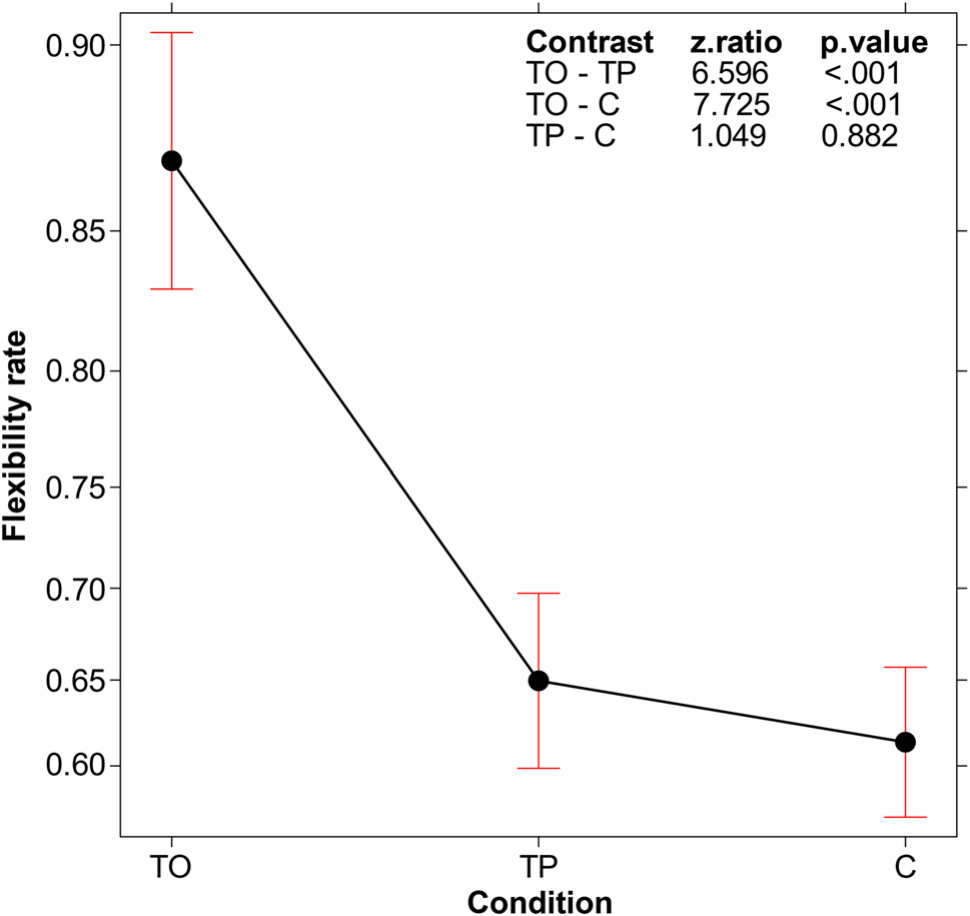
Main effect of condition on flexibility (operationalized as the number of test triples focusing on different aspects of the seed triple over the total number of test triples produced). Error bars represent the 95% confidence interval

We expressed the degree of flexibility proportionally, in terms of the number of test triples that focused on different aspects over the total number of test triples produced. This implied that a higher value indicated a greater degree of flexibility. A GLM was subsequently used on these values (logit link function, binomial family), with condition as the fixed effect and participants as the random effect. Condition was found to be significant (*χ*^*2*^(2, *N* = 180) = 62.124, *p* < .001). As shown in Fig. 4, flexibility was higher in the TO condition, compared to both TP and C.

### e) Critical dimensions

Two independent judges (two of the experimenters) classified the test triples produced by each participant by identifying whether or not they tested the critical dimension of ascending-descending (by submitting at least one descending test triple), the dimension of constant-variable intervals (by submitting at least one triple with numbers separated by variable intervals), and the dimension of “odd-even” numbers (by submitting at least one triple including one or more odd numbers). In all cases, the coding was yes or no (i.e., 0 –1). The inter-rater reliability agreement between the two judges, measured by Cohen’s kappa coefficient, was good (never less than 0.86).

Three GLMs (log link function, Poisson family) were conducted to analyze the mean frequency of triples produced by each participant in the test phase. These GLMs examined the frequency of triples fitting the criteria of three critical dimensions across the three experimental conditions. In each GLM condition was treated as the fixed effect, and participants was treated as the random effect.

**Fig. 5:**
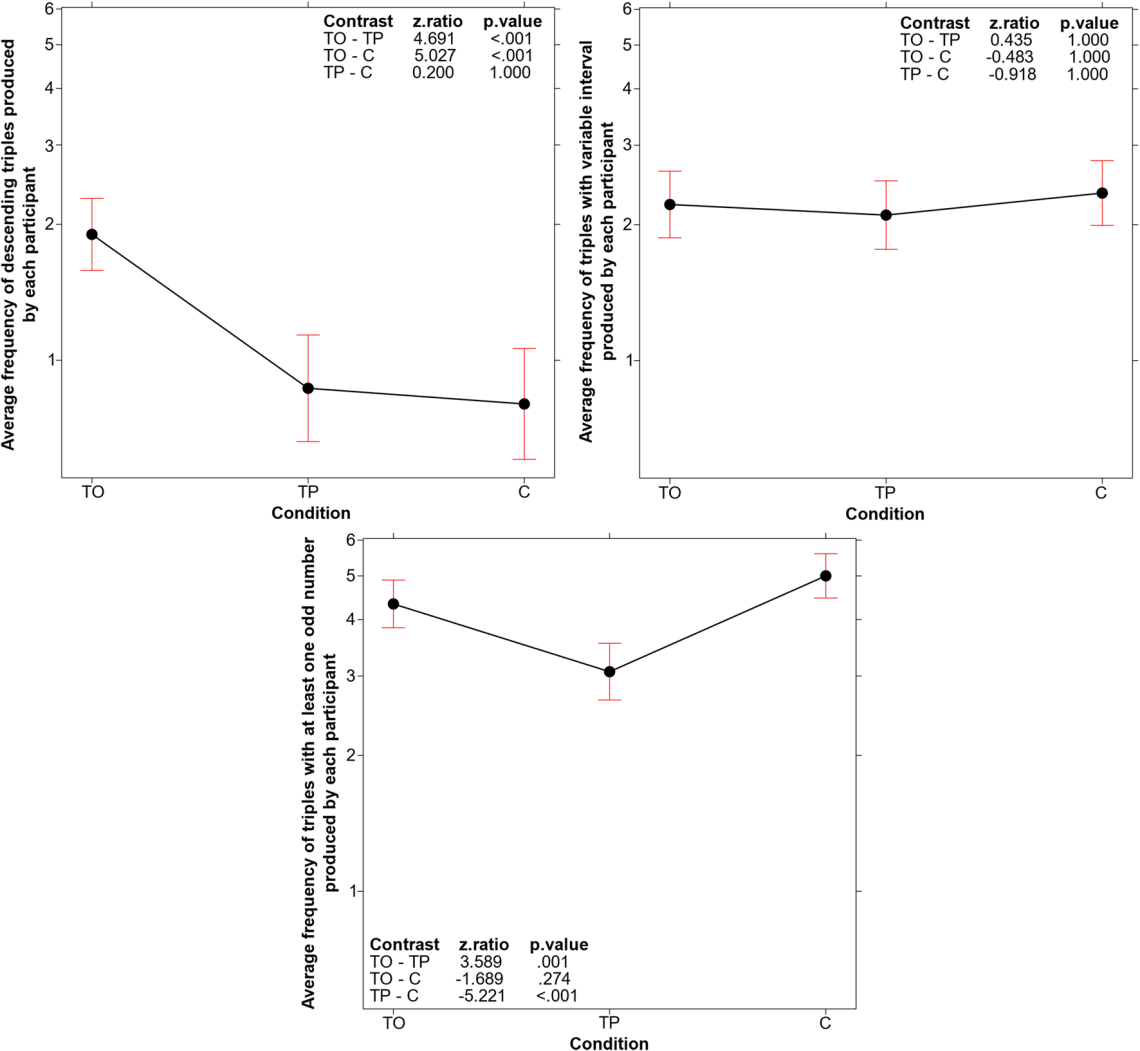
A **Top left panel:** Main effect of condition on the average frequency of descending triples produced by each participant. **Top right panel:** Main effect of condition on the average frequency of triples with a variable interval between numbers produced by each participant. **Bottom panel:** Main effect of condition on the average frequency of triples with at least one odd number produced by each participant. Error bars represent the 95% confidence interval

A significant main effect of condition was found for the submission of descending triples (*χ*^*2*^(2, *N* = 180) = 35.986, *p* < .001). As shown in Fig. 5 (top left panel), the average frequency of descending triples was significantly higher in the TO condition compared to the other two conditions. Conversely (see Fig. 5, top right panel), no significant difference was observed in the average frequency of triples that tested the variability of intervals between the numbers within the triple across the three conditions (*χ*^*2*^(2, *N* = 180) = 0.8443, *p* = .655). The average frequency of triples testing the dimension of odd-even numbers (Fig. 5, bottom panel) was higher in both the TO and C conditions compared to the TP condition (*χ*^*2*^(2, *N* = 180) = 28.938, *p* < .001).

Therefore, what seems to be specifically associated with the TO condition is the tendency of participants to identify the critical property (ascending-descending) and test it by using a descending triple.

We further investigated the use of descending triples by conducting a GLM (log link function, Poisson family). The response variable was the total number of participants (out of 60 in each condition) who submitted a descending triple. Condition (TO, TP, and C) and attempt (first, second, or third) were included as fixed effects.

**Fig. 6:**
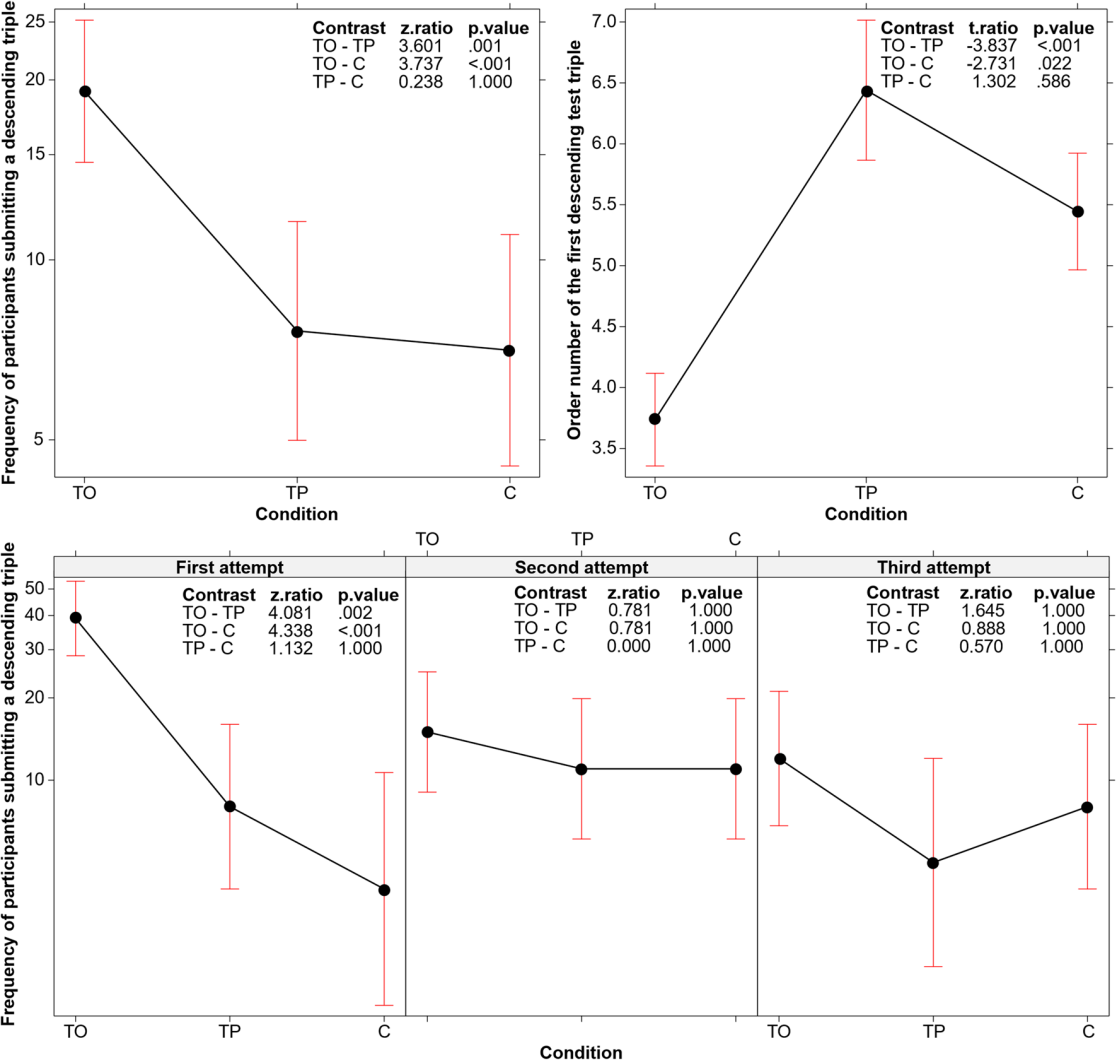
A** Top left panel:** Number of participants submitting a descending test triple in TO, TP, and C conditions. **Top right panel:** Order number of the first descending test triple submitted in the three conditions. **Bottom panel:** Number of participants submitting a descending test triple for each attempt separately (interaction between condition and attempt). In all graphs, error bars represent the 95% confidence interval

The main effect of condition was confirmed (*χ*^*2*^(2, *N* = 180) = 29.710, *p* < .001), as shown in the top left panel of Fig. 6. However, a significant interaction between condition and attempt also emerged (*χ*^*2*^(4, *N* = 180) = 15.249, *p* = .004), indicating that the difference between the three conditions concerned the first attempt (see Fig. 6, bottom panel). Specifically, the frequency of participants who tested the ascending-descending triple before their first attempt at finding the solution was higher in TO than in TP and C.

Another way to explore the time the participants took to consider the critical property (ascending-descending) involved analyzing the order number of the first descending triple submitted (i.e., whether it was, for instance, the third or the 11th triple in the list of triples submitted). This analysis involved the subset of participants who actually produced a descending triple. The order number of the descending triple was coded by listing all the test triples produced during the experiment by each participant and assigning the corresponding order number to the first descending triple that appeared in the list. Subsequently, an LM was conducted on the order number, with condition as the fixed effect. As shown in the top right panel of Fig. 6, an ascending-descending triple appeared after a greater number of test triples in the TP and the C conditions, compared to the TO condition (*F*(2, *N* = 115) = 8.491, *p* < 0.001).

## General discussion

This study investigates the effects of contrast/opposites on overcoming default biases in inductive reasoning and adds to the limited existing studies on this topic (Branchini et al., 2023; Gale & Ball, 2012; Gorman & Gorman, 1984; Gorman et al., 1984, 1987; Rossi et al., 2001). The rationale behind expecting that a general prompt to think in opposites could be both easily applicable and potentially beneficial in various reasoning contexts has been outlined recently in two review papers concerning reasoning processes in general (Branchini et al., 2021) and creativity in particular (Bianchi & Branchini, 2023). However, a solid empirical basis of evidence is needed to clarify the strength, generalizability, and limits of this hypothesis. This research contributes to developing such an empirical basis of evidence by adding new data regarding the effects of a prompt to think in opposites while engaged in a hypothesis-testing task (namely Wason’s rule-discovery task). Since hypothesis testing is a reasoning process pervading everyday life, exploring the effect of the prompt in this type of reasoning process is meaningful and important, especially because people have an intuitive understanding of what opposites are, making the strategy accessible to anyone.

Our findings demonstrate that the suggestion is indeed beneficial. Participants trained to address the task by using opposites were significantly more successful in solving it. This finding supports the results of a previous study (Branchini et al., 2023), which, however, did not clarify whether the positive effect concerned the use of opposites per se or the analytical focus on the properties of the seed triple included in the training (specifically, the first step of the training). Our study enabled us to clarify this aspect as we designed two training conditions besides a no-training control condition. In the training based on listing the triple’s properties (TP), participants were recommended to follow a strategy involving two steps: they were prompted to identify all the properties characterizing the original triple (first step) and encouraged to use the identified properties to produce the test triples (second step). In the training based on opposites (TO), after the instruction to identify all of the properties characterizing the original triples (first step, identical to TP), participants were invited to identify the opposite of each property listed (second step) and use these opposites to produce the test triples (third step).

The TP training did not improve participants’ performance. In contrast, thinking in terms of opposites after identifying the properties characterizing the seed triple significantly impacted participants’ success. Almost 80% of the participants in the TO condition found a solution, which is almost twice the proportion of participants who found it in the TP and C conditions (see Fig. 1). This high success rate was particularly evident in the participants’ first attempt to articulate the rule (see Fig. 2, bottom graph).

The aspect that stimulated this efficacy improvement was revealed by our analyses of the *type of search process* activated by the TO participants. The TO condition was not associated with a higher number of test triples submitted (Fig. 3), indicating that the better performance depended not on the participants’ persistence in testing triples, but rather on the efficacy of the tests they conducted. Indeed, we found that participants in the TO condition were more flexible in their testing strategies compared to the two other conditions (see Fig. 4); that is, the training stimulated them to avoid reiterating assessments of the same hypothesis and to extend their solution search space in various directions. Particularly, the analyses of the three critical aspects to be tested (not ascending, but descending triples; not regular, but irregular intervals; not even, but odd numbers) demonstrated that participants in the TO condition specifically differed from those in the other conditions by more frequently testing descending triples (Figs. 5 and 6, top left panel). They did so sooner than those in the other two conditions, in terms of the order number in the series of triples tested (see Fig. 6, top right panel and bottom panel). In other words, the TO participants recognized the critical dimension and used them before and more often than those in the other two conditions.

The application of the training (both TP and TO) incurred a cost in terms of the time needed to find the solution. This is not surprising, as there was additional work required of the participants – i.e., to list the properties focused on in TP and additionally, their opposites in TO. This identification and enunciation were done before submitting each test triple. Future research might want to separate the time needed to identify and list the properties (and their opposites) from the time concerning the testing phase *in the strict sense* (i.e., the time occupied by the submission of the test triples). However, although technically feasible, it is debatable whether the phase of identification and listing of the properties (and their opposites) does not already belong to the “solution search phase.” We feel it does, and hence, in the present study, we counted as “solution time” the total time from the presentation of the seed triple to the discovery of the rule (exactly as in previous studies where the training was applied to visuo-spatial problem solving, and the time needed to list all the spatial properties of the problem and their relative opposites was considered part of the solution process phase – see Branchini et al., 2015, 2016; Bianchi et al., 2020).

In some studies on hypothesis testing (e.g., Augustinova, 2008) participants were ensured the same amount of elaboration time before generating the hypothesis to be tested in all studied conditions. As a development of the present study, it might be worthwhile to consider the effect of constraining participants to generate a triple after a fixed period of time, for example, 90-s intervals, in all three conditions (TO, TP, and C). This would guarantee the same amount of elaboration time before submitting each triple and would make it possible to rule out elaboration time as a potential confounding factor. Another worthwhile manipulation of our three conditions might involve constraining the number of triples to be submitted before enunciating the rule, such as limiting it to ten triples for all three conditions (as in Gale & Ball, 2012). We have found that participants in the C condition produced more triples than in the other two (TP and TO). This constraint might stimulate them to reduce the quantity of the triples submitted and instead focus on their quality and flexibility.

Our findings regarding success rates and change in the aspects focused on during the solution process parallel the broader picture that emerged when a prompt to explore the structure of the problem in terms of opposites was given concerning visuo-spatial problems. In the latter case, the prompt not only led to greater success than control groups (Bianchi et al., 2020; Branchini et al., 2015, 2016) but also resulted in a modification in the participants’ approach to the problem. An analysis of the verbal interactions within the groups (Branchini et al., 2015) revealed that participants were more focused on a careful analysis of the perceptual structure of the problem and on finding perceptual solutions, rather than relying on notions and formulas they might have had in mind. An analysis of the drawings created in the attempt to solve the problems revealed that participants exhibited less fixedness and operated within a more extended search space (Branchini et al., 2016).

Returning to hypothesis testing (and Wason’s task) and considering Oaksford and Chater’s iterative counterfactual model (1994a, b), according to which the behavior of a hypothetical reasoning subject leads them to generate and test both working and counterfactual hypotheses, we observed that the training to think in opposites facilitated the assumption of a counterfactual strategy, leading to more effective exploration of the boundaries of the rule’s search space. Counterfactual hypotheses are those for which the reasoner (prone to find confirmation for an idea they have in mind) expects to receive negative feedback. Thinking counterfactually is challenging, as we discussed in the *Introduction*, but thinking in opposites facilitates the process, possibly because it still allows for the application of a confirmatory strategy. In fact, if the participant focuses on the hypothesis “sequence of *even* numbers in *increasing* order,” the prompt to think in opposites stimulates them to concentrate not on even but on *odd numbers*. Once this shift occurs (and reasoners are no longer focused on even numbers), they assume the opposite property as the target of their reasoning and submit a triple comprising odd numbers with the aim of testing (and confirming) it is wrong. Similarly, when they focus on the “increasing” property, the prompt to think in opposites encourages them to concentrate not on increasing numbers but on *decreasing numbers,* thus consistently submitting a descending test triple aimed at confirming it is wrong. In both cases, they expect negative feedback. If they receive positive feedback instead, they will be compelled to revise their hypothesis, rather than perseverate on it.

The prompt, in other words, enables reasoners to see both sides of the dimension (white and black swans), rather than only one property (white swans). The effect is similar to what Gale and Ball (2012) pursued in the dual task when they highlighted the critical dimension by offering 2–4-6 as an example of the DAX rule and 6-4-2 as one of the MED rules. This pair of triples makes the two poles of the dimension noticeable (they are not only theoretically available but also perceivable). Similarly, in our task, naming the properties and their opposites makes the dimension perceivable. The benefit in terms of solution rates found in the two cases (around 75% in Gale & Ball, 2012; around 78% in our study) is similar.

A limitation of this study is that as it is focused exclusively on Wason’s task, generalizations of the conclusions to hypothesis testing in general are not tenable at this stage, and require further exploration. It would be interesting to see future studies assessing the applicability of the “thinking in opposites” strategy in a range of real-world domains in which extending the boundaries of the search space would be directly beneficial to hypothesis testing performance, for example, in interpersonal contexts (where hypotheses about a person’s characteristics are drawn), concerning social prejudice (where hypotheses about a group are tested), or in medical contexts (where diagnostic hypotheses are made). We hope this study might inspire interest in pursuing these new research paths.

## Data Availability

The data set used for this article can be accessed at https://dx.doi.org/10.21227/s9dq-7852.

## Appendix 1

### Training based on opposites (TO)

I suggest a strategy that can be used to generate your test triples. This strategy will help you discover the rule. To explain it, I will refer to triples made up of shapes, but in the task, numbers are used.

A triple set of shapes that conforms to the rule I have in mind is given below. Look carefully at the three shapes and consider the properties they have in common.

For instance:

- All the sides are straight.
- They are all plane figures (2D).
- They are all symmetrical figures.
- They are all closed figures.
- The figures are aligned with one another.
- They are all empty figures.
- They all have different shapes (i.e. they are different from one another).

Since you have to discover the rule I have in mind, you need to figure out which of these characteristics is relevant to the rule. How can this be done? I suggest “thinking in opposites,” which means focusing on one characteristic at a time and identifying its opposite. For instance, if all the shapes have *straight* sides, you might ask yourself whether this characteristic is crucial. To verify this, carry out a test with a triple set composed of shapes that have *curved* sides. If the feedback indicates that the triple you have proposed is still a good example of the rule, this means that it is irrelevant whether the shapes have straight or curved sides.

You should then focus on another characteristic and test it similarly, e.g., they are all *bi-dimensional* shapes. To verify whether this feature is crucial, generate a triple with *three-dimensional* shapes, and if this is still a good example of the rule I have in mind, it means that this too is an irrelevant characteristic.

Another characteristic to test might be that all the shapes are *symmetrical*. If you generate a triple set with *asymmetrical* shapes, and it is still a good example of the rule I have in mind, it again means that this is an irrelevant characteristic.

I hope you have understood what to do. If so, I will now show you the triple set of numbers that represents the rule you are asked to find. Focus on one characteristic of the set at a time, write it down, and then think of its opposite; that is, find out whether a triple set that is opposite in terms of the characteristic you are focusing on still conforms to the rule. Write this test triple (and the feedback you receive) on your response sheet. You can test as many triples as you like, noting them in the order that you test them. Once you think you have identified the rule, say it aloud (and write it too), and I will tell you whether it is correct. If it is not, you can continue to test using other triples but remember that you have only three chances to guess the rule. Are you ready? The test triple is 2-4-6.

### Training based on listing the triple’s properties (TP)

I suggest to you a strategy that can be used to generate your test triples. It will help you to discover the rule. To explain it, I will refer to triples made up of shapes, but in the task, numbers are used.

A triple set of shapes that conforms to the rule I have in mind is given below. Look carefully at the three shapes and consider the properties they have in common.

For instance:

- All the sides are straight.
- They are all plane figures (2D).
- They are all symmetrical figures.
- They are all closed figures.
- The figures are aligned with one another.
- They are all empty figures.
- They all have different shapes (i.e. they are different from one another).

Since you have to discover the rule I have in mind, you need to figure out which of these characteristics is relevant to the rule. How can this be done? I suggest focusing on one characteristic at a time. For instance, if all the shapes have *straight* sides, you might ask yourself whether this characteristic is crucial.

You should then focus on another characteristic; for example, they are all *bi-dimensional* shapes, and you might ask yourself whether this characteristic is crucial.

Another characteristic to test might concern the fact that all the shapes are *symmetrical,* and you might ask yourself whether this characteristic is crucial.

I hope you have understood what to do. If so, I will now show you the triple set of numbers that represents the rule you are asked to find. Focus on one characteristic of the set at a time, write it down and then test a triple set with the characteristic you are focusing on. I will give you feedback regarding its conformity to the rule. Write this test triple (and the feedback you receive) on your response sheet. You can test as many triples as you like, noting them in the order you test them. Once you think you have identified the rule, say it aloud (and write it), and I will tell you whether it is correct. If it is not, you can continue to test using other triples but remember that you have only three chances to guess the rule. Are you ready? The test triple is 2-4-6.

## Code availability

The script for R code used for this article can be accessed at https://dx.doi.org/10.21227/s9dq-7852.
